# Supplementary material for: Suspected Frostbite Injuries in Coypu (Myocastor coypus)
Source: Animals (Basel). 2022 Oct 14;12(20):2777. doi: 10.3390/ani12202777 (PMC9597802; doi:10.3390/ani12202777)
Supplement: Supplementary file 1 [file animals-12-02777-s001.zip › animals-1959353-supplementary.pdf]

Case Report

Suspected Frostbite Injuries in Coypu (*Myocastor coypus*)

Friederike Gethöffer [1,*,†], Katharina M. Gregor [2†], Isabel Zdora [2], Peter Wohlsein [2], Franziska Schöttes [1] and Ursula Siebert [1]

[1] Institute for Terrestrial and Aquatic Wildlife Research, University of Veterinary Medicine Hannover, Foundation, Bischofsholer Damm 15, 30173 Hannover, Germany
[2] Department of Pathology, University of Veterinary Medicine Hannover, Foundation, Bünteweg 17, 30559 Hannover, Germany
* Correspondence: friederike.gethöffer@tiho-hannover.de;
† Contribution of authors equally first.

Supplementary Materials

Table S1. Additional pathological findings in ten coypus (*Myocastor coypus*) from Lower Saxony, Germany.

Table S2. Daily temperatures in different levels above ground from DWD – stations during a 10 days period prior to nearby harvesting of the animals (source: https://cdc.dwd.de/rest/metadata/station/xy)

Table S1. Additional pathological findings in ten coypus (*Myocastor coypus*) from Lower Saxony, Germany.

Observation	*Case N°*	*Animals (n = 10)*
**Respiratory system**		
Pulmonary edema	1, 2, 3, 4, 6, 8, 9, 10	8
Pulmonary emphysema	1, 3, 4, 6, 8, 10	6
Lymphoplasmacytic pneumonia	2, 3, 8	3
Granulomatous pneumonia	5	1
**Alimentary system**		
Hepatitis	8	1
**Urogenital system**		
Urolithiasis	4, 8, 10	3
**Endocrine system**		
Vacuolation of zona fasciculata	7, 8, 9	3
**Hematopoietic system**		
Lymphonodular follicular hyperplasia	3	1
Lymphonodular hemosiderosis	3, 7	2
Lymphonodular anthracosis	7	1
Spleenic hemosiderosis	1, 4, 5	3

Table S2. Daily temperatures in different levels above ground from DWD – stations during a 10 days period prior to nearby harvesting of the animals (source: https://cdc.dwd.de/rest/metadata/station/xy).

Date	Ahaus			Emden			Göttingen			Hannover			Wolfsburg		
	TG	TN	TM	TG	TN	TM	TG	TN	TM	TG	TN	TM	TG	TN	TM
01.01.2021	0.4 °C	1.7 °C	2.7 °C	0.3 °C	1.6 °C	2.7 °C	0.7 °C	1.9 °C	3.2 °C	-1.5 °C	1.9 °C	4.7 °C	-0.2 °C	2.2 °C	4.9 °C
02.01.2021	1.6 °C	2.7 °C	3.6 °C	1 °C	2.5 °C	3.7 °C	-1.3 °C	0.5 °C	3 °C	0.3 °C	2.4 °C	4.3 °C	0.7 °C	2.7 °C	4.8 °C
03.01.2021	1.3 °C	1.9 °C	3 °C	1.2 °C	2.7 °C	3.4 °C	-0.4 °C	0.5 °C	1.4 °C	0.1 °C	1.1 °C	2.1 °C	0.4 °C	1.7 °C	2.9 °C
04.01.2021	1.2 °C	1.7 °C	2 °C	0.9 °C	2.2 °C	3.2 °C	0.5 °C	0.8 °C	1.4 °C	0.9 °C	1.3 °C	1.8 °C	0.5 °C	0.9 °C	1.4 °C
05.01.2021	0.4 °C	1.2 °C	1.9 °C	1.2 °C	2 °C	2.5 °C	0.1 °C	1 °C	1.8 °C	0.5 °C	1.5 °C	2.8 °C	0.6 °C	1.8 °C	3.2 °C
06.01.2021	0.2 °C	0.8 °C	1.3 °C	0 °C	0.8 °C	1.6 °C	-0.5 °C	0.8 °C	1.7 °C	0.2 °C	1.1 °C	2.5 °C	0.3 °C	1.4 °C	2.7 °C
07.01.2021	0.2 °C	0.9 °C	1.5 °C	0.5 °C	1.1 °C	1.8 °C	-0.3 °C	0.4 °C	1.4 °C	0.4 °C	1 °C	2 °C	0.1 °C	0.7 °C	1.6 °C
08.01.2021	-0.3 °C	0.7 °C	1.6 °C	-0.3 °C	1.3 °C	2.6 °C	-0.7 °C	0.4 °C	1.4 °C	-0.7 °C	0.6 °C	1.9 °C	-0.4 °C	0.8 °C	1.4 °C
09.01.2021	-0.6 °C	0.6 °C	2.2 °C	-1.5 °C	0.1 °C	2.6 °C	-1.3 °C	0 °C	1.1 °C	-2 °C	-0.4 °C	1.7 °C	-0.4 °C	0.1 °C	0.6 °C
10.01.2021	-0.1 °C	1.5 °C	2.9 °C	-0.7 °C	1.2 °C	2.7 °C	-2.1 °C	-0.4 °C	2.4 °C	-1.5 °C	0.9 °C	3.4 °C	-1.3 °C	0.7 °C	2.7 °C
11.01.2021	2.7 °C	3.6 °C	5.3 °C	2.8 °C	3.8 °C	5.7 °C	-0.2 °C	0.6 °C	2 °C	1.5 °C	2.5 °C	4.3 °C	0.9 °C	1.7 °C	2.6 °C
12.01.2021	1.8 °C	4.9 °C	6.3 °C	1.5 °C	4.2 °C	6.5 °C	0.5 °C	1.7 °C	3.4 °C	1.1 °C	3.6 °C	6 °C	1.4 °C	2.3 °C	3.6 °C
13.01.2021	1.7 °C	2.9 °C	4.7 °C	0.6 °C	2.9 °C	5.4 °C	-0.1 °C	1 °C	2.3 °C	0 °C	1.8 °C	3.4 °C	-0.1 °C	2.1 °C	3.8 °C
14.01.2021	-0.9 °C	0.2 °C	1.5 °C	-0.8 °C	0.4 °C	1.6 °C	-3.3 °C	-0.3 °C	1.5 °C	-0.7 °C	0.7 °C	2.4 °C	0.1 °C	0.7 °C	1.9 °C
15.01.2021	-2.2 °C	-0.7 °C	0.8 °C	-2.5 °C	-0.3 °C	0.6 °C	-4.2 °C	-1.1 °C	-0.1 °C	-3.1 °C	-0.8 °C	0.3 °C	-2.3 °C	-0.6 °C	0.4 °C
16.01.2021	-2.3 °C	-0.7 °C	1.3 °C	-1.5 °C	0 °C	1.2 °C	-4.4 °C	-2 °C	-0.2 °C	-4.3 °C	-2.1 °C	-0.5 °C	-2.7 °C	-1.6 °C	-0.3 °C
17.01.2021	-0.5 °C	0.8 °C	2.6 °C	-0.7 °C	0 °C	2 °C	-3.3 °C	-1.8 °C	-0.1 °C	-2.3 °C	-1 °C	0.8 °C	-3.4 °C	-1.6 °C	0.3 °C
18.01.2021	1.9 °C	3.4 °C	4.8 °C	0.1 °C	2.8 °C	4.2 °C	-0.5 °C	1.9 °C	3.1 °C	0.3 °C	2.6 °C	4.1 °C	-1 °C	2.9 °C	4.6 °C
19.01.2021	4.5 °C	6.4 °C	8.6 °C	3.1 °C	5.8 °C	8.5 °C	1.8 °C	3.8 °C	7 °C	3.5 °C	5.4 °C	8.8 °C	3 °C	4.4 °C	8.3 °C
20.01.2021	7.7 °C	8.7 °C	9.9 °C	6.4 °C	7.5 °C	8.7 °C	2.6 °C	5.7 °C	8.2 °C	5.5 °C	7.7 °C	9.2 °C	4.4 °C	7.5 °C	10.1 °C
21.01.2021	7.2 °C	8.8 °C	9.6 °C	5.3 °C	7.2 °C	9.5 °C	5.2 °C	7.7 °C	8.9 °C	5.7 °C	7.8 °C	9.6 °C	6.7 °C	8.6 °C	12.2 °C
22.01.2021	2.9 °C	5.3 °C	8 °C	1.1 °C	3.9 °C	5.7 °C	0.2 °C	4.9 °C	9.1 °C	0.2 °C	5.3 °C	9.4 °C	0.4 °C	6 °C	11.3 °C
23.01.2021	1.7 °C	3 °C	5.2 °C	0.1 °C	1.8 °C	4.9 °C	0.1 °C	1.6 °C	4.9 °C	-0.6 °C	1.9 °C	5.9 °C	-0.1 °C	2.4 °C	6.3 °C
24.01.2021	-0.2 °C	1.2 °C	3 °C	-0.9 °C	0.8 °C	3.4 °C	-0.2 °C	0.6 °C	1.5 °C	-0.7 °C	0.5 °C	1.7 °C	0.2 °C	1.4 °C	2.3 °C
25.01.2021	-1.7 °C	0.5 °C	2.9 °C	-1.5 °C	0.5 °C	2.7 °C	-2.9 °C	-0.6 °C	2.2 °C	-2.2 °C	-0.1 °C	3.1 °C	-1.2 °C	0.9 °C	3.7 °C
26.01.2021	1.6 °C	2.8 °C	4.9 °C	0.1 °C	1.8 °C	4.4 °C	-2 °C	-0.1 °C	2.2 °C	-0.5 °C	0.9 °C	3.7 °C	-0.1 °C	1 °C	2.4 °C
27.01.2021	1.4 °C	2.6 °C	4.5 °C	0.2 °C	1.5 °C	4.1 °C	-1.6 °C	0.3 °C	2.6 °C	-0.6 °C	1.3 °C	4.1 °C	0.2 °C	2 °C	3.7 °C
28.01.2021	1.4 °C	3 °C	4.2 °C	-0.1 °C	1.3 °C	3.1 °C	0.8 °C	2.6 °C	3.7 °C	-0.5 °C	2.7 °C	4.6 °C	0 °C	2.8 °C	4.8 °C
29.01.2021	1.4 °C	3.6 °C	6.2 °C	-1.1 °C	-0.1 °C	0.3 °C	0.3 °C	2.9 °C	6.9 °C	-1.3 °C	-0.3 °C	0.5 °C	-1.3 °C	-0.1 °C	0.8 °C

30.01.2021	-2.3 °C	0 °C	3.1 °C	-4.7 °C	-1.4 °C	0.2 °C	-2.3 °C	0 °C	5.5 °C	-6 °C	-1.5 °C	0.7 °C	-4.6 °C	-1.2 °C	0.4 °C
31.01.2021	-4.4 °C	-2 °C	0.8 °C	-8.5 °C	-4 °C	1.1 °C	-4.4 °C	-2 °C	2.3 °C	-6.4 °C	-3.8 °C	1.4 °C	-9.9 °C	-5.4 °C	-0.3 °C
01.02.2021	-2 °C	-0.5 °C	0.8 °C	-5.1 °C	-3.2 °C	-1.3 °C	-3.1 °C	0.2 °C	3.7 °C	-5.2 °C	-1.5 °C	1.3 °C	-6.4 °C	-2 °C	0.7 °C
02.02.2021	-0.3 °C	0.7 °C	3.2 °C	-2.3 °C	-0.7 °C	0.3 °C	0.1 °C	2.5 °C	7.4 °C	-0.8 °C	0.6 °C	2.1 °C	0.4 °C	1.1 °C	2.2 °C
03.02.2021	3.8 °C	6.3 °C	8.7 °C	0 °C	2.8 °C	5.7 °C	5.5 °C	7.6 °C	9.8 °C	1.2 °C	5.9 °C	9 °C	1.5 °C	6.3 °C	10 °C
04.02.2021	3.7 °C	5.4 °C	6.6 °C	0.3 °C	0.9 °C	1.8 °C	2.6 °C	6.1 °C	8.2 °C	2.4 °C	4.9 °C	6.9 °C	1.5 °C	2.9 °C	6.5 °C
05.02.2021	2.6 °C	3.7 °C	5.2 °C	0.2 °C	1 °C	2.1 °C	1.8 °C	3.5 °C	5 °C	1.1 °C	2.2 °C	4.1 °C	0.1 °C	1.8 °C	2.6 °C
06.02.2021	***-1.1 °C***	***1.1 °C***	***3.6 °C***	***-2.1 °C***	***-0.6 °C***	***0.3 °C***	***-1.3 °C***	***1.6 °C***	***3.5 °C***	***-2.1 °C***	***-0.6 °C***	***0.8 °C***	***-3.5 °C***	***-1.2 °C***	***0.1 °C***
07.02.2021	***-6.6 °C***	***-4.7 °C***	***-1.1 °C***	***-6.1 °C***	***-4.8 °C***	***-2.5 °C***	***-6.4 °C***	***-5.1 °C***	***-1.7 °C***	***-6.7 °C***	***-5.6 °C***	***-2.4 °C***	***-6.9 °C***	***-6 °C***	***-3.5 °C***
08.02.2021	***-7.5 °C***	***-6.5 °C***	***-5.6 °C***	***-5.6 °C***	***-4.5 °C***	***-2.8 °C***	***-10.2 °C***	***-8.8 °C***	***-6.5 °C***	***-9.5 °C***	***-8.2 °C***	***-6.5 °C***	***-9.8 °C***	***-8.2 °C***	***-6.1 °C***
09.02.2021	***-7.6 °C***	***-6.1 °C***	***-4.2 °C***	***-6.2 °C***	***-3.4 °C***	***-0.9 °C***	***-15.5 °C***	***-11.8 °C***	***-8 °C***	***-15 °C***	***-10.1 °C***	***-6.1 °C***	***-12.1 °C***	***-9.8 °C***	***-7.4 °C***
10.02.2021	***-7.2 °C***	***-5.5 °C***	***-3.4 °C***	***-6 °C***	***-3.7 °C***	***-1 °C***	***-19.2 °C***	***-11.2 °C***	***-4.6 °C***	***-17.2 °C***	***-9 °C***	***-1.8 °C***	***-13.1 °C***	***-7.5 °C***	***-2.7 °C***
11.02.2021	***-10.3 °C***	***-6.9 °C***	***-2.9 °C***	***-7.8 °C***	***-4.1 °C***	***-0.5 °C***	***-11 °C***	***-6.8 °C***	***-4.1 °C***	***-13.6 °C***	***-9.2 °C***	***-3.2 °C***	***-11 °C***	***-6.7 °C***	***-2.7 °C***
12.02.2021	***-9.6 °C***	***-7.4 °C***	***-4.9 °C***	***-8.4 °C***	***-5.3 °C***	***-1.5 °C***	***-17.2 °C***	***-11.3 °C***	***-3.9 °C***	***-16.7 °C***	***-9.3 °C***	***-2.5 °C***	***-12.8 °C***	***-7.8 °C***	***-3.3 °C***
13.02.2021	***-11.5 °C***	***-7.4 °C***	***-3 °C***	***-10.8 °C***	***-6.2 °C***	***-0.8 °C***	***-20.6 °C***	***-13.4 °C***	***-5.6 °C***	***-18.3 °C***	***-11.5 °C***	***-5.1 °C***	***-18.7 °C***	***-11.9 °C***	***-4.3 °C***
14.02.2021	***-10 °C***	***-4.2 °C***	***2.1 °C***	***-9.9 °C***	***-4.3 °C***	***1.2 °C***	***-19.8 °C***	***-10.2 °C***	***-0.9 °C***	***-14.7 °C***	***-8.6 °C***	***-1.4 °C***	***-17.6 °C***	***-10.4 °C***	***-2.1 °C***
15.02.2021	***-1.8 °C***	***0.8 °C***	***4.2 °C***	***-1.3 °C***	***0.5 °C***	***2.7 °C***	***-6.6 °C***	***-2.5 °C***	***2.1 °C***	***-4.7 °C***	***-1.1 °C***	***4 °C***	***-8 °C***	***-2.2 °C***	***1.7 °C***
16.02.2021	3.5 °C	6.1 °C	8.6 °C	2.5 °C	4.8 °C	7.6 °C	1.8 °C	3.4 °C	6.3 °C	2.4 °C	4.3 °C	7.6 °C	1.5 °C	3.7 °C	6.6 °C
17.02.2021	6.7 °C	8 °C	9.5 °C	5.6 °C	6.6 °C	7.9 °C	2.5 °C	4.7 °C	7.9 °C	3.9 °C	6.2 °C	8.9 °C	4.2 °C	6.6 °C	9.1 °C
18.02.2021	6.7 °C	8.9 °C	11.8 °C	4.1 °C	7.7 °C	10.3 °C	1.1 °C	5.1 °C	9.8 °C	2.7 °C	6.6 °C	10.8 °C	4 °C	7.5 °C	12 °C

* TG = Minimum temperature 5 cm above ground, TN = Minimum temperature 2 m above ground, TM = mean temperature 2 m above ground, period of consecutive severe frost days in bold italics.
